# Stress hyperglycemia ratio: an independent predictor for in-hospital major adverse cardiovascular and cerebrovascular events in patients with st-segment elevation myocardial infarction

**DOI:** 10.1186/s12872-023-03219-6

**Published:** 2023-04-15

**Authors:** Wen Guo, Jiajia Zhu, Wenxian Liu

**Affiliations:** Department of Cardiology, Beijing Anzhen Hospital, Capital Medical University, No. 2 Anzhen Road, Chaoyang District, Beijing, 100029 China

**Keywords:** Diabetes mellitus, MACCE, Stress hyperglycemia ratio, ST-segment elevation myocardial infarction

## Abstract

**Background:**

To assess the predictive accuracy of the stress hyperglycemia ratio (SHR) for in-hospital major adverse cardiovascular and cerebrovascular events (MACCE) in patients with ST-segment elevation myocardial infarction (STEMI).

**Methods:**

A total of 1,944 patients were enrolled within 24 h of a new STEMI diagnosis. The SHR was obtained by dividing the blood glucose level at admission by the estimated average glucose. MACCE were defined as acute cerebral infarction, mechanical complications of myocardial infarction, cardiogenic shock, and all-cause death. Patients were then categorized into the MACCE and non-MACCE groups according to the occurrence of in-hospital MACCE. Propensity score matching was used to balance confounding factors, and logistic regression was used to identify the potential predictive factors for MACCE.

**Results:**

A total of 276 patients were included after 1:1 matching, and the confounding factors were balanced between the two groups. The SHR was an independent predictor of in-hospital MACCE (odds ratio = 10.06, 95% confidence interval: 4.16–27.64, *P* < 0.001), while blood glucose at admission was not. The SHR was also an independent predictor for in-hospital MACCE in nondiabetic patients with STEMI (odds ratio = 11.26, 95% confidence interval: 3.05–55.21, *P* < 0.001).

**Conclusion:**

SHR is an independent predictor of in-hospital MACCE in patients with acute STEMI, especially in nondiabetic patients.

## Background

ST-segment elevation myocardial infarction (STEMI) is the most severe type of coronary heart disease. With advances in primary prevention, interventional therapy, and drug therapy, the incidence of STEMI has declined in developed countries. However, it remains a leading cause of death worldwide [1]. A previous study reported that diabetes mellitus (DM), one of the well-recognized risk factors for coronary heart disease, increases the morbidity and mortality rates of patients with acute myocardial infarction (AMI) [2]. Subsequent studies identified stress hyperglycemia as a predictor of prognosis for AMI patients [3, 4]. In 2015, Roberts et al*.* proposed replacing conventional blood glucose levels with the stress hyperglycemia ratio (SHR) to predict the severity of critical illness [5]. Another study reported that the SHR is closely associated with the short-term prognosis of AMI patients [6]. However, it remains unclear whether the SHR, as a monitoring parameter, can be used as an independent predictor in patients with STEMI.

In the present study, we aimed to evaluate the predictive value of the SHR for in-hospital major adverse cardiovascular and cerebrovascular events (MACCE) in patients with newly diagnosed STEMI within the previous 24 h. As the high predictive value of stress hyperglycemia for the prognosis of AMI may be related to its effects on myocardial metabolism and insulin treatment for DM [7, 8], we also evaluated the predictive potential of the SHR in STEMI patients without diabetes (nondiabetic subgroup).

## Methods

### Study participants

**Fig. 1:**
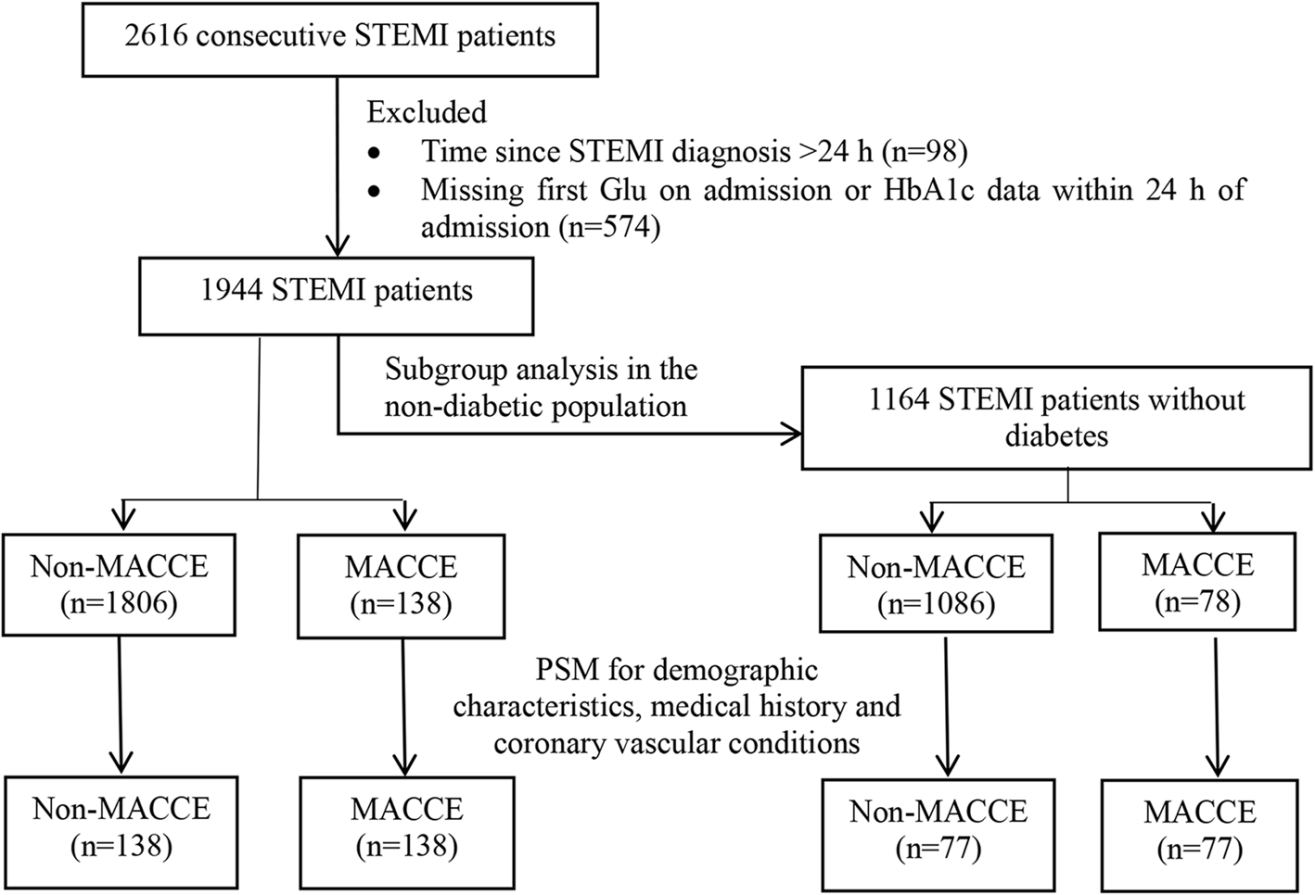
Flowchart of patient enrollment and subgroup analysis

In this retrospective study, 2,616 patients who were diagnosed with STEMI in our hospital from January 1st, 2015, to December 31st, 2019, were consecutively screened. The inclusion criteria were as follows: (1) met the diagnostic criteria for STEMI [9] and (2) aged over 18 years. Patients were excluded if: (1) STEMI was diagnosed more than 24 h previously or (2) blood glucose levels at admission (Glu) or glycated hemoglobin (HbA1c) levels were not measured within 24 h of admission. Finally, 1,944 patients were recruited. The process of recruitment is shown in Fig. 1. This study was approved by the Ethics Committee of Beijing Anzhen Hospital, Capital Medical University (Approval number: 2022170X). All patients provided written informed consent.

Because only 7.1% of the enrolled patients had poor outcomes, 1:1 nearest neighbor propensity score matching (PSM) was used to control possibly biased baseline covariates. The propensity score was estimated using a multivariate logistic regression model. As gender, age, and history of hypertension, diabetes, chronic kidney disease, and smoking, as well as coronary vascular conditions are independent risk factors for poor prognosis of STEMI [1], the demographic characteristics, medical history, and coronary vascular conditions of patients were included in the PSM model for 1:1 matching.

### Definitions

STEMI was diagnosed if there was a fall and/or rise in the cardiac troponin (cTn) level, with at least one value above the 99th percentile upper reference limit and at least one of the following symptoms: development of pathological Q waves, new ST-segment elevation on electrocardiogram, myocardial ischemia-related symptoms, and imaging evidence of new regional wall motion abnormality or new loss of viable myocardium in a pattern consistent with ischemic etiology [9].

The SHR was defined as the Glu divided by the estimated average glucose derived from HbA1c [5]. The following formula was used: SHR = Glu (within 24 h of STEMI onset) / [(1.59 × HbA1c) – 2.59].

The main observational outcome was in-hospital MACCE, defined as acute ischemic stroke, mechanical complications of myocardial infarction (MI), cardiogenic shock, and all-cause death.

Patients with no previously diagnosed DM or a HbA1c level of less than 6.5% were considered nondiabetic [10].

### Data collection

The demographic characteristics and data regarding the history of smoking, hypertension, dyslipidemia, chronic renal disease, stroke, and coronary revascularization of all patients were collected from medical records based on standardized definitions by two experienced data inspectors. The number of significantly narrowed vessels was counted as any coronary arteries with ≥ 50% stenosis in the three main branches without considering left main artery disease. The following clinical data were also collected: diastolic blood pressure (DBP) and systolic blood pressure (SBP) at admission, respiratory rate, heart rate (HR), Killip class, occurrence of arrhythmia (new atrial or ventricular arrhythmias after STEMI), usage of intra-aortic balloon pump (IABP), ventilator, blood platelet count, white blood cell (WBC) count, and levels of hemoglobin, alanine transaminase, albumin, aspartate aminotransferase, C-reactive protein (CRP), serum creatinine (Cr), Glu, HbA1c, high-density lipoprotein cholesterol, low-density lipoprotein cholesterol, total triglycerides, total cholesterol, brain natriuretic peptide (BNP), creatine kinase, lactate dehydrogenase, cardiac troponin I, and serum potassium. All blood samples were analyzed immediately after collection in our medical center. The left ventricular ejection fraction (LVEF) and left ventricular end-diastolic dimension were measured by echocardiography using Simpson’s method.

### Statistical analysis

Continuous variables are expressed as mean ± standard deviation or median (25th percentile, 75th percentile) according to the results of the normality test. Differences in continuous data between two groups were assessed by independent sample *t*-test or Mann–Whitney U test. Categorical data are shown as number (percentage). Differences in categorical variables between two groups were analyzed by χ^2^ or Fisher’s exact test.

Patients in the MACCE group were matched to those in the non-MACCE group using a structured, iterative propensity score model, with the primary objective of maximizing the balance in the distribution of possible confounders between the two groups. Then, PSM was performed separately for all patients and the nondiabetic subgroup, which included all baseline data, such as age, gender, medical history, and coronary vascular conditions. The corresponding propensity score of the variables was calculated for each patient in the MACCE group. A nearest neighbor matching algorithm was used to match patients in the MACCE group with those in the non-MACCE group at a 1:1 ratio (with no replacement) within 0.2 × standard deviation of the logit of the propensity score. To determine whether PSM achieved balance in all potential confounders, all baseline characteristics after PSM were compared between the groups.

To screen independent risk factors for patient prognosis, indicators with statistical significance after PSM were included in the multivariate logistic regression analysis using forward selection. *P* < 0.05 was considered statistically significant. R version 4.2.1 (R Foundation for Statistical Computing, Vienna, Austria) was used to conduct all statistical analyses and to prepare all graphical presentations.

## Results

### Baseline data

A total of 1,944 patients who were diagnosed with STEMI were enrolled. The baseline characteristics of these patients are presented in Table 1. The mean age of this cohort was 58.43 years. Among them, 1505 (77.4%) were male and 1,689 (86.9%) underwent percutaneous coronary intervention (PCI). During hospitalization, 138 (7.1%) patients experienced MACCE, including 28 (1.4%) deaths. The SHR of patients who experienced MACCE was higher than that of those who did not experience MACCE (*P* < 0.05).

**Table 1:** Demographic characteristics, medical history, and coronary vascular conditions of all patients before and after propensity score matching (PSM)

Variables	Before PSM				After PSM			
	**Non-MACCE** **(*n* = 1806)**	**MACCE** **(*n* = 138)**	**t/χ2**	***P*-value**	**Non-MACCE** **(*n* = 138)**	**MACCE** **(*n* = 138)**	**t/χ2**	***P*-value**
Age	58.21 ± 11.83	61.36 ± 2.14	2.971	0.003^*^	61.36 ± 13.96	61.36 ± 12.14	0.005	0.996
Gender (male)	1403 (77.7%)	102 (73.9%)	1964.760	0.36	105 (76.1%)	102 (73.9%)	256.569	0.781
**Medical history**								
Hypertension	992 (54.9%)	83 (60.1%)	1980.397	0.272	72 (52.2%)	83 (60.1%)	292.407	0.225
Smoking	1131 (62.6%)	86 (62.3%)	1793.182	> 0.99	86 (62.3%)	86 (62.3%)	221.719	> 0.99
Dyslipidemia	231 (12.8%)	25 (18.1%)	2024.040	0.098	27 (19.6%)	25 (18.1%)	247.947	0.878
CKD	145 (8.0%)	31 (22.46%)	2148.302	< 0.001^*^	30 (21.7%)	31 (22.5%)	219.631	> 0.99
Prior Stroke	157 (8.7%)	18 (13.0%)	2017.455	0.117	16 (11.6%)	18 (13.0%)	250.294	0.855
Prior PAD	26 (1.4%)	2 (1.4%)	1768.430	< 0.99	1 (0.7%)	2 (1.4%)	215.928	> 0.99
Prior PCI	192 (10.6%)	13 (9.4%)	1898.249	0.762	12 (8.7%)	13 (9.4%)	199.563	> 0.99
Prior CABG	12 (0.7%)	2 (1.4%)	1927.067	0.597	2 (1.4%)	2 (1.4%)	209.719	> 0.99
**Coronary vascular condition**								
Primary PCI	1576 (87.3%)	113 (81.9%)	2025.545	0.094	109 (79.0%)	113 (81.9%)	265.470	0.649
LM	120 (6.6%)	20 (14.5%)	2148.579	0.001^*^	19 (13.8%)	20 (14.5%)	218.623	> 0.99
LAD	1284 (71.1%)	100 (72.5%)	1888.804	0.807	96 (69.6%)	100 (72.5%)	262.820	0.691
LCX	810 (44.9%)	67 (48.6%)	1950.018	0.451	65 (47.1%)	67 (48.6%)	244.903	0.904
RCA	918 (50.8%)	72 (52.2%)	1883.711	0.829	74 (53.6%)	72 (52.2%)	244.903	0.904
Multi-vessels	1564 (86.6%)	113 (81.9%)	2006.296	0.155	74 (53.6%)	113 (81.9%)	265.470	0.649

*PSM* Propensity score matching, *MACCE* Major adverse cardiovascular and cerebrovascular events, *CKD* Chronic kidney disease, *PAD* Peripheral arterial disease, *PCI* Percutaneous coronary intervention, *CABG* Coronary artery bypass grafting, *LM* left main coronary artery, *LAD* Left anterior descending coronary artery, *LCX* Left circumflex coronary artery, *RCA* Right coronary artery

^*^P < 0.05

The gender and history of smoking, hypertension, dyslipidemia, stroke, peripheral arterial disease, coronary artery bypass grafting, and PCI were comparable between the MACCE and non-MACCE groups. Patients in the MACCE group were older and had a higher rate of chronic kidney disease than those in the non-MACCE group. The rate of primary PCI and the incidence rates for disease involving the right coronary artery, left circumflex coronary artery, left anterior descending coronary artery, and multiple vessels were comparable between the two groups. The incidence of left main coronary artery disease was higher in the MACCE group than in the non-MACCE group. Meanwhile, patients who experienced MACCE also showed a higher HR, respiratory rate, Killip class, occurrence of arrhythmia, and usage of IABP and ventilator, but lower blood pressure (both SBP and DBP), albumin level, total triglyceride level, and LVEF than those who did not experience MACCE.

**Fig. 2:**
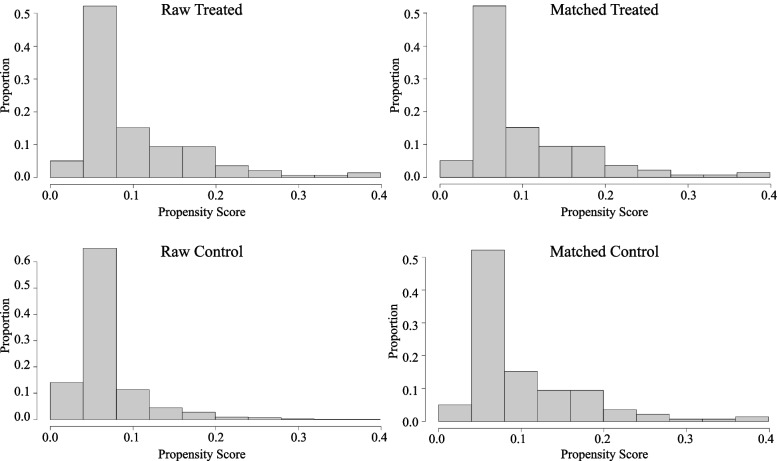
Clinical features of STEMI patients before and after propensity score matching

PSM was then performed to eliminate confounding factors. STEMI patients in the MACCE and non-MACCE groups (*n* = 138 per group), with no significant difference in demographic characteristics, medical history, or coronary vascular conditions, were further analyzed. After matching, the SHR of the MACCE group was higher than that of the non-MACCE group (1.00 ± 0.30 *vs.* 1.18 ± 0.43, *P* < 0.001). Moreover, patients in the MACCE group showed a significantly higher HR, Killip class, occurrence of arrhythmia, usage of IABP and ventilator, WBC, Glu, CRP, and Cr, but lower SBP, DBP, HBG, and LVEF than those in the non-MACCE group (*P* < 0.05) (Fig. 2, Tables 1–2).

**Table 2:** Clinical characteristics and biochemical variables of all STEMI patients before and after PSM

Variables	Before PSM					After PSM			
	**Non-MACCE** **(*n* = 1806)**	**MACCE** **(*n* = 138)**		**t/χ2**	***P*-value**	**Non-MACCE** **(*n* = 138)**	**MACCE** **(*n* = 138)**	**t/χ2**	***P*-value**
**Clinical characteristics**									
SBP (mmHg)	120.27 ± 20.53		103.77 ± 26.64	3.456	< 0.001^*^	121.86 ± 22.91	103.77 (26.64)	3.360	< 0.001^*^
DBP (mmHg)	73.54 ± 12.87		64.88 ± 16.32	3.365	< 0.001^*^	72.86 ± 12.67	64.88 ± 16.32	3.477	< 0.001^*^
HR (bpm)	76.82 ± 15.31		88.42 ± 22.69	3.307	< 0.001^*^	79.54 ± 17.50	88.42 ± 22.69	3.394	< 0.001^*^
RR (bpm)	18.54 ± 3.04		19.57 ± 3.78	3.328	< 0.001^*^	18.88 ± 3.09	19.57 ± 3.78	1.655	0.099
Killip III&IV	63 (3.5%)		52 (37.7%)	2154.697	< 0.001^*^	11 (8.0%)	52 (37.7%)	359.095	< 0.001^*^
Arrhythmia	65 (3.6%)		28 (20.3%)	2149.143	< 0.001^*^	6 (4.3%)	28 (20.3%)	353.539	< 0.001^*^
IABP	253 (14.0%)		83 (60.1%)	2141.750	< 0.001^*^	24 (17.4%)	83 (60.1%)	353.625	< 0.001^*^
Ventilator	32 (1.8%)		34 (24.6%)	2149.143	< 0.001^*^	7 (5.1%)	34 (24.6%)	353.711	< 0.001^*^
**Biochemical variables**									
WBC (× 10^9^/L)	9.63 ± 3.39		11.95 ± 4.59	3.498	< 0.001^*^	9.96 ± 3.90	11.95 ± 4.59	3.377	< 0.001^*^
HGB (g/L)	136.95 ± 17.19		138.46 ± 18.91	0.991	0.322	132.02 ± 19.47	138.46 ± 18.91	2.769	0.006^*^
PLT (× 10^9^/L)	208.65 ± 60.17		208.08 ± 57.03	0.108	0.914	201.89 ± 51.95	208.08 ± 57.03	0.942	0.347
ALT (U/L)	53.75 ± 127.33		143.15 ± 517.96	3.446	< 0.001^*^	59.39 ± 147.29	143.15 ± 517.96	1.819	0.070
ALB (g/L)	38.90 ± 4.17		37.60 ± 4.72	3.296	0.001^*^	37.61 ± 4.58	37.97 ± 5.66	0.575	0.566
AST (U/L)	141.88 ± 227.76		240.94 ± 522.43	3.365	< 0.001^*^	144.69 ± 283.99	240.94 ± 522.43	1.896	0.059
Cr (μmo1/L)	85.06 ± 40.97		101.55 ± 60.88	3.985	< 0.001^*^	103.84 ± 49.17	101.55 ± 60.88	0.343	0.732
Glu (mmol/L)	7.70 ± 3.03		9.03 ± 3.44	3.328	< 0.001^*^	7.21 ± 2.91	9.03 ± 3.44	3.370	< 0.001^*^
HbA1c (%)	6.54 ± 1.52		6.58 ± 1.50	0.331	0.741	6.61 ± 1.50	6.58 ± 1.50	0.170	0.865
SHR	1.00 ± 0.30		1.18 ± 0.43	3.399	< 0.001^*^	0.92 ± 0.28	1.18 ± 0.43	3.394	< 0.001^*^
TG (mmol/L)	1.75 ± 1.13		1.45 ± 1.15	2.971	0.003^*^	1.62 ± 0.95	1.45 ± 1.15	1.296	0.196
TC (mmol/L)	4.50 ± 1.04		4.33 ± 1.20	1.752	0.080	4.36 ± 1.02	4.33 ± 1.20	0.192	0.848
HDL-C (mmol/L)	1.01 ± 0.28		1.03 ± 0.30	0.754	0.451	0.97 ± 0.26	1.03 ± 0.30	1.586	0.114
LDL-C (mmol/L)	2.83 ± 0.86		2.70 ± 0.95	1.707	0.088	2.76 ± 0.82	2.70 ± 0.95	0.553	0.581
CRP (mg/L)	11.87 ± 11.54		17.72 ± 13.77	3.498	< 0.001^*^	13.73 ± 12.23	17.72 ± 13.77	2.401	0.017^*^
BNP (ng/L)	760.38 ± 1584.71		1051.89 ± 2181.27	1.101	0.271	883.94 ± 1342.41	1051.89 ± 2181.27	0.505	0.614
cTnI (ng/ml)	36.97 ± 82.01		45.75 ± 93.43	1.193	0.233	38.06 ± 72.39	45.75 ± 93.43	0.758	0.449
CK (U/L)	1097.85 ± 1518.40		1581.33 ± 2547.54	3.296	0.001^*^	980.20 ± 1417.10	1581.33 ± 2547.54	2.424	0.016^*^
LDH (U/L)	448.59 ± 404.61		537.39 ± 563.50	2.411	0.016^*^	456.44 ± 538.40	537.39 ± 563.50	1.221	0.223
K (mmol/L)	4.06 ± 0.46		4.08 ± 0.52	0.711	0.477	4.06 ± 0.43	4.08 ± 0.52	0.417	0.677
LVEDD (mm)	49.78 ± 5.88		50.34 ± 7.68	1.011	0.312	50.30 ± 5.70	50.34 ± 7.68	0.051	0.959
LVEF (%)	54.45 ± 12.9		47.98 ± 11.29	3.403	< 0.001^*^	52.27 ± 11.71	47.98 ± 11.29	2.994	0.003^*^

*PSM* Propensity score matching, *MACCE* Major adverse cardiovascular and cerebrovascular events, *SBP* Systolic blood pressure, *DBP* Diastolic blood pressure, *HR* Heart rate, *RR* Respiratory rate, *IABP* Intra-aortic balloon pump, *WBC* White blood cell, *HGB* Hemoglobin, *PLT* Blood platelet count, *ALT* Alanine transaminase, *ALB* Albumin, *AST* Aspartate aminotransferase, *Cr* Serum creatinine, *Glu* first blood glucose on admission within 24 h, *HbA1c* Glycosylated hemoglobin, *SHR* Stress hyperglycemia ratio, *TG* Total triglycerides, *TC* Total cholesterol, *HDL-C* High-density lipoprotein cholesterol, *LDL-C* Low-density lipoprotein cholesterol, *CRP* C-reactive protein, *BNP* Brain natriuretic peptide, *cTnI* Cardiac troponin I, *CK* Creatine kinase, *LDH* Lactate dehydrogenase, *K* Serum potassium, *LVEDD* Left ventricular end-diastolic dimension, *LVEF* Left ventricular ejection fraction

^*^P < 0.05

### Predictive value of SHR for in-hospital outcomes in patients with STEMI

**Fig. 3:**
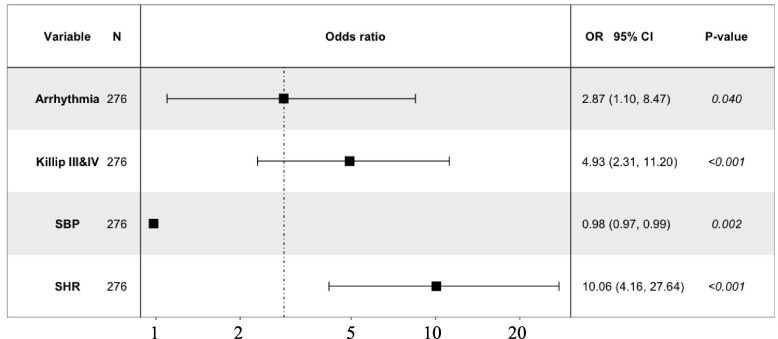
Multivariate logistic regression analysis of predictors of in-hospital outcomes among all STEMI patients after PSM

The 138 cases with MACCE included 28 cases of all-cause death, 5 cases of acute ischemic stroke, 21 cases with mechanical complications of MI, and 95 cases of cardiogenic shock. After PMS matching, three factors related to in-hospital MACCE were included in the multivariate logistic regression analysis: SHR (odds ratio [OR] = 10.06, 95% confidence interval [CI]: 4.16–27.64, *P* < 0.001), occurrence of arrhythmia (OR = 2.87, 95% CI: 1.10–8.47, *P* = 0.040), and acute heart failure (OR = 4.93, 95% CI: 2.31–11.20, *P* < 0.001). They all were independent risk factors for in-hospital MACCE. SBP was a protective factor for in-hospital MACCE in patients with STEMI (OR = 0.98, 95% CI: 0.97–0.99, *P* = 0.002), whereas Glu was not an independent risk factor for MACCE (Table 3, Fig. 3).

**Table 3:** Multivariate logistic regression analysis of predictors of in-hospital outcomes in STEMI patients after PSM

Variables	β	S.E	OR	95% Cl	*P-*value
Arrhythmia	1.05	0.51	2.87	1.10–8.47	0.040*
Killip III&IV	1.60	0.40	4.93	2.31–11.20	< 0.001*
SBP	-0.02	0.96	0.98	0.97–0.99	0.002*
SHR	2.31	0.48	10.06	4.16–27.64	< 0.001*

*S.E.* Standard error, *OR* Odds ratio, *CI* Confidence interval, *SBP* Systolic blood pressure, *SHR* Stress hyperglycemia ratio

^*^P < 0.05

### Predictive value of SHR in nondiabetic patients

**Fig. 4:**
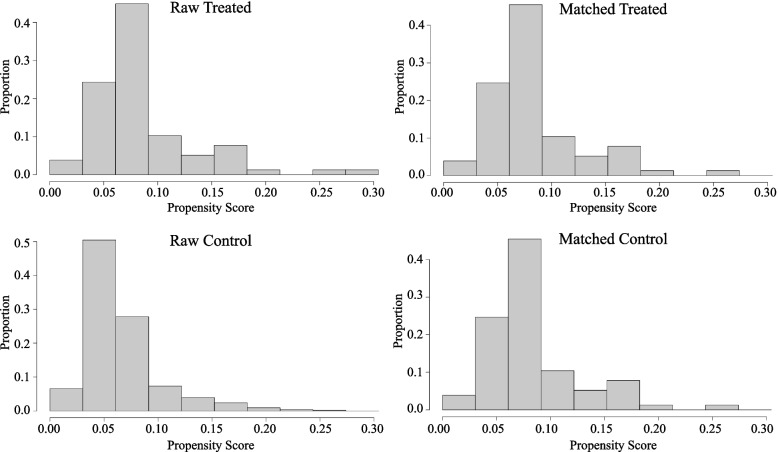
Clinical features of nondiabetic STEMI patients before and after PSM

To eliminate the effects of long-term hyperglycemia on the metabolism of patients with DM and the effects of regular use of insulin and other hypoglycemic drugs on the prognosis, subgroup analysis was performed in people without DM. Before PSM, the non-MACCE and MACCE groups included 1,086 and 78 nondiabetic patients, respectively. After PSM, the non-MACCE and MACCE groups included 77 nondiabetic patients each. No significant difference in baseline characteristics, including age, gender, medical history, and coronary vascular conditions, was found between the two groups. The SHR remained higher in the MACCE group than in the non-MACCE group (0.99 ± 0.23 vs 1.24 ± 0.45, *P* < 0.001). The HR, occurrence with Killip III&IV and arrhythmia, usage of IABP and ventilator, WBC, Glu, CRP, and Cr in the MACCE group were significantly higher, while the SBP, DBP, HBG, TC, and LVEF were significantly lower than those in the non-MACCE group (*P* < 0.05) (Table 4, Fig. 4).

**Table 4:** Characteristics of STEMI patients without diabetes before and after PSM

Variables	Before PSM				After PSM			
	Non-MACCE (*n* = 1086)	MACCE (*n* = 78)	**t/χ2**	*P*-value	Non-MACCE (*n* = 77)	MACCE (*n* = 77)	**t/χ2**	*P*-value
Age	57.04 ± 11.96	59.95 ± 12.70	2.066	0.039^*^	60.48 ± 13.80	59.78 ± 12.69	0.328	0.743
Gender (male)	878 (80.8%)	60 (76.9%)	1944.678	0.485	53 (68.8%)	60 (77.9%)	163.058	0.274
**Medical history**								
Hypertension	560 (51.6%)	38 (48.7%)	1907.687	0.712	34 (44.2%)	38 (49.4%)	146.707	0.628
Smoking	716 (65.9%)	48 (61.5%)	1941.396	0.506	43 (55.8%)	48 (62.3%)	151.810	0.512
Dyslipidemia	128 (11.8%)	12 (15.4%)	1950.966	0.445	11 (14.3%)	12 (15.6%)	107.477	> 0.99
CKD	74 (6.8%)	11 (14.1%)	2053.498	0.04^*^	7 (9.1%)	10 (13.0%)	147.645	0.607
Prior Stroke	81 (7.5%)	6 (7.7%)	1768.430	> 0.99	6 (7.8%)	6 (7.8%)	110.623	> 0.99
Prior PAD	11 (1.0%)	2 (2.6%)	1944.991	0.483	2 (2.6%)	1 (1.3%)	113.385	> 0.99
Prior PCI	89 (8.2%)	4 (5.1%)	1949.545	0.454	4 (5.2%)	4 (5.2%)	107.477	> 0.99
**Coronary vascular condition**								
Primary PCI	966 (89.0%)	67 (85.9%)	1938.740	0.523	66 (85.7%)	67 (87.0%)	111.698	> 0.99
LM	67 (6.2%)	10 (12.8%)	2052.755	0.041^*^	8 (10.4%)	10 (13.0%)	138.000	0.802
LAD	768 (70.7%)	57 (73.1%)	1899.822	0.754	52 (67.5%)	57 (74.0%)	153.299	0.478
LCX	768 (70.7%)	57 (73.1%)	1899.822	0.754	46 (59.7%)	40 (51.9%)	156.020	0.417
RCA	542 (49.9%)	36 (46.2%)	1926.426	0.601	38 (49.4%)	36 (46.8%)	133.365	0.872
Multi-vessels	958 (88.2%)	67 (85.9%)	1915.385	0.668	66 (85.7%)	67 (87.0%)	112.602	> 0.99
**Clinical characteristics**								
SBP (mmHg)	119.74 ± 19.81	103.13 ± 25.49	3.395	< 0.001^*^	121.44 ± 21.83	103.17 ± 25.65	3.620	< 0.001^*^
DBP (mmHg)	73.88 ± 12.93	65.00 ± 14.95	3.325	< 0.001^*^	73.86 ± 13.73	65.06 ± 15.04	3.386	< 0.001^*^
HR (bpm)	75.76 ± 14.55	90.72 ± 25.16	3.622	< 0.001^*^	76.39 ± 15.16	90.62 ± 25.31	3.620	< 0.001^*^
RR (bpm)	18.37 ± 2.85	19.56 ± 4.20	3.296	0.001^*^	18.78 ± 3.19	19.51 ± 4.19	1.210	0.228
Killip III&IV	28 (2.6%)	27 (34.6%)	2148.302	< 0.001^*^	2 (2.6%)	27 (35.1%)	215.075	< 0.001^*^
Arrhythmia	33 (3.0%)	19 (24.4%)	2158.858	< 0.001^*^	1 (1.3%)	19 (24.7%)	214.228	< 0.001^*^
IABP	124 (11.4%)	43 (55.1%)	2151.251	< 0.001^*^	13 (16.9%)	43 (55.8%)	217.180	< 0.001^*^
Ventilator	10 (0.9%)	16 (20.5%)	2164.131	< 0.001^*^	2 (2.6%)	16 (20.8%)	212.797	0.001^*^
**Biochemical variables**								
WBC (× 10^9^/L)	9.50 ± 3.22	12.61 ± 4.68	3.487	< 0.001^*^	9.48 ± 3.60	12.59 ± 4.70	3.505	< 0.001^*^
HGB (g/L)	138.13 ± 16.35	139.30 ± 19.30	0.604	0.546	133.23 ± 17.62	139.46 ± 19.37	2.093	0.038^*^
PLT (× 10^9^/L)	209.29 ± 60.05	204.74 ± 51.87	0.653	0.514	207.98 ± 50.27	206.58 ± 49.58	0.174	0.862
ALT (U/L)	52.01 ± 84.69	78.31 ± 192.27	2.328	0.02*	49.51 ± 47.79	56.97 ± 43.97	0.994	0.322
ALB (g/L)	39.12 ± 4.02	37.75 ± 4.80%	2.882	0.004^*^	38.88 ± 4.46	37.91 ± 5.30	1.195	0.234
AST (U/L)	138.19 ± 183.38	209.12 ± 385.82	2.971	0.003^*^	155.63 ± 170.20	173.26 ± 221.85	0.552	0.582
Cr (μmo1/L)	82.66 ± 34.85	97.28 ± 62.62	3.296	0.001	91.18 ± 74.45	95.57 ± 61.17	0.400	0.69
Glu (mmol/L)	6.45 ± 1.66	8.00 ± 2.90	3.358	< 0.001^*^	6.39 ± 1.47	7.99 ± 2.92	3.421	< 0.001^*^
HbA1c (%)	5.67 ± 0.38	5.71 ± 0.35	1.018	0.309	5.73 ± 0.41	5.71 ± 0.34	0.424	0.672
SHR	1.01 ± 0.27	1.24 ± 0.45	3.622	< 0.001^*^	0.99 ± 0.23	1.24 ± 0.45	3.460	< 0.001^*^
TG (mmol/L)	1.67 ± 0.97	1.30 ± 0.61	3.296	0.001	1.69 ± 1.08	1.30 ± 0.61	2.646	0.009^*^
TC (mmol/L)	4.51 ± 1.03	4.36 ± 1.13	1.216	0.224	4.44 ± 1.02	4.37 ± 1.14	0.394	0.694
HDL-C (mmol/L)	1.02 ± 0.27	1.08 ± 0.30	1.651	0.099	1.03 ± 0.22	1.08 ± 0.30	1.123	0.263
LDL-C (mmol/L)	2.83 ± 0.86	2.73 ± 0.96	0.991	0.322	2.78 ± 0.82	2.74 ± 0.96	0.260	0.795
CRP (mg/L)	10.76 ± 10.86	19.33 ± 13.59	3.437	< 0.001^*^	11.34 ± 10.71	19.37 ± 13.68	3.505	< 0.001^*^
BNP (ng/L)	556.41 ± 1125.30	1180.39 ± 2927.63	2.110	0.035	624.00 ± 882.37	503.12 ± 578.03	0.514	0.608
cTnI (ng/ml)	34.88 ± 64.52	53.55 ± 111.09	2.310	0.021	52.39 ± 92.32	52.63 ± 111.52	0.015	0.988
CK (U/L)	1139.23 ± 1580.77	1455.87 ± 1994.59	1.675	0.094	1226.78 ± 1756.01	1442.91 ± 2004.36	0.710	0.479
LDH (U/L)	440.49 ± 357.39	517.42 ± 534.29	1.763	0.078	452.22 ± 337.43	485.36 ± 456.07	0.511	0.61
K (mmol/L)	4.05 ± 0.45	4.02 ± 0.52	0.590	0.555	4.08 ± 0.39	4.01 ± 0.53	0.895	0.372
LVEDD (mm)	49.63 ± 5.72	4.02 ± 0.52	0.590	0.555	49.04 ± 4.71	49.28 ± 7.57	0.216	0.829
LVEF (%)	55.40 ± 12.31	47.62 ± 10.68	3.437	< 0.001^*^	55.72 ± 9.97	47.77 ± 10.68	3.700	< 0.001^*^

*PSM* Propensity score matching, *MACCE* Major adverse cardiovascular and cerebrovascular events, *CKD* Chronic kidney disease, *PAD* Peripheral arterial disease, *PCI* Percutaneous coronary intervention, *CABG* Coronary artery bypass grafting, *LM* Left main coronary artery, *LAD* Left anterior descending coronary artery, *LCX* Left circumflex coronary artery, *RCA* Right coronary artery, *SBP* Systolic blood pressure, *DBP* Diastolic blood pressure, *HR* Heart rate, *RR* Respiratory rate, *IABP* Intra-aortic balloon pump, *WBC* White blood cell, *HGB* Hemoglobin, *PLT* Blood platelet count, *Alt* Alanine transaminase, *ALB* Albumin, *AST* Aspartate aminotransferase, *Cr* Serum creatinine, *Glu* First blood glucose on admission within 24 h, *HbA1c* Glycosylated hemoglobin, *SHR* Stress hyperglycemia ratio, *TG* Total triglycerides, *TC* Total cholesterol, *HDL-C* High-density lipoprotein cholesterol, *LDL-C* Low-density lipoprotein cholesterol, *CRP* C-reactive protein, *BNP* Brain natriuretic peptide, *cTnI* Cardiac troponin I, *CK* Creatine kinase, *LDH* Lactate dehydrogenase, *K* serum potassium, *LVEDD* Left ventricular end-diastolic dimension, *LVEF* Left ventricular ejection fraction

^*^P < 0.05

**Fig. 5:**
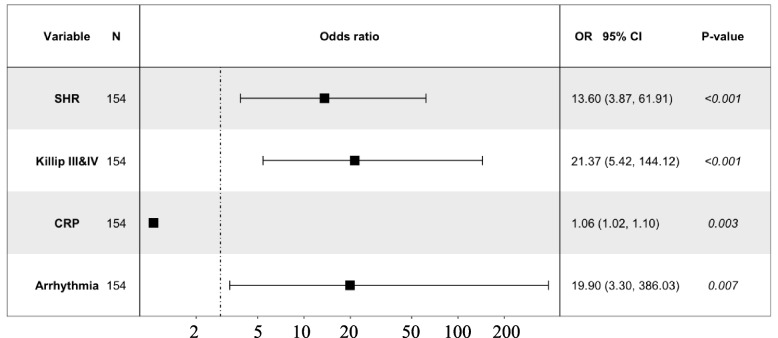
Multivariate logistic regression analysis of predictors of in-hospital outcomes in the nondiabetic subgroup after PSM

Among nondiabetic patients, 78 cases experienced MACCE, including 14 cases of all-cause death, 2 cases of acute ischemic stroke, 11 cases with mechanical complications of MI, and 57 cases of cardiogenic shock. After PMS matching, 77 cases of MACCE were included, including 13 cases of all-cause death, 2 cases of acute ischemic stroke, 10 cases with mechanical complications of MI, and 57 cases of cardiogenic shock. Four factors related to in-hospital MACCE in patients with STEMI were included in the multivariate logistic regression analysis, including the SHR (OR = 11.26, 95% CI: 3.05–55.21, *P* < 0.001), occurrence of arrhythmia (OR = 16.79, 95% CI: 2.72–328.46, *P* = 0.011), acute heart failure (OR = 14.35, 95% CI: 3.47–98.88, *P* = 0.001), and CRP (OR = 1.05, 95% CI: 1.02–1.09, *P* = 0.003). All four factors were found to be independent risk factors for in-hospital MACCE in the nondiabetic subgroup. However, Glu was not an independent risk factor for in-hospital MACCE in this subgroup (Table 5, Fig. 5).

**Table 5:** Multivariate logistic regression analysis of predictors of in-hospital outcomes in STEMI patients without diabetes after PSM

Variables	β	S.E	OR	95%Cl	*P*-value
Arrhythmia	2.82	1.11	16.79	2.72–328.46	0.011^*^
Killip III&IV	2.66	0.82	14.35	3.47–98.88	0.001^*^
SHR	2.42	0.73	11.26	3.05–55.21	< 0.001^*^
CRP	0.05	0.18	1.05	1.02–1.09	0.003^*^

*S.E*. Standard error, *O*: Odds ratio, *CI* Confidence interval, *SHR* Stress hyperglycemia ratio, *CRP* C-reactive protein

^*^P < 0.05

## Discussion

In this study, STEMI patients were recruited independent of the occurrence of MACCE, and age, cardiac function, renal function, and coronary vascular conditions were included in a PSM model. Our findings suggest that the SHR is an independent predictor for in-hospital MACCE in this population.

Assessment of the prognostic value of several inexpensive and readily available laboratory variables, such as serum glucose level, in STEMI is important. In 2009, Horne et al. from the Intermountain Medical Center developed the Intermountain Risk Score (IMRS) to evaluate the overall health status and estimate the mortality risk from all causes in the general population [11]. Several studies have shown that the IMRS can predict mortality in patients with STEMI or STEMI accompanied by cardiogenic shock [12, 13].

Furthermore, stress hyperglycemia is associated with adverse outcomes in AMI [14]. Previous studies have shown that the blood glucose level is an independent risk factor for in-hospital death in patients with cardiogenic shock [15, 16]. A positive correlation between stress hyperglycemia and intracoronary thrombotic burden has been reported [17]. Khalfallah et al*.* found that stress hyperglycemia is associated with increased incidences of cardiogenic shock, contrast-induced nephropathy, and no-reflow phenomenon, as well as higher mortality, in nondiabetic STEMI patients undergoing primary PCI [6]. Furthermore, hyperglycemia during perioperative coronary revascularization leads to increased inflammatory platelet activity and endothelial dysfunction, which may be related to plaque instability [18]. A positive correlation between hyperglycemia at admission and MI size has also been observed in patients with STEMI [19, 20]. The above data suggest that stress hyperglycemia may indicate the severity of acute disease and be predictive of prognosis.

Patients with STEMI are under stress conditions, which induce the secretion of a large amount of cortisol hormones, such as epinephrine and catecholamines, from the adrenal cortex, resulting in a sharp rise in blood glucose in a short period of time. Previous evidence demonstrated that stress hyperglycemia promotes the production of free fatty acids, which aggravates oxidative stress, endothelial damage, and inflammation, and increases cortisol hormone and blood glucose levels [21]. Endothelial dysfunction, microcirculation impairment, thrombosis, and stress hyperglycemia all contribute to the poor prognosis of patients with STEMI [22, 23]. Thus, stress hyperglycemia may contribute to worse short-term outcomes in patients with higher glucose levels but without previously diagnosed DM.

Stress hyperglycemia has been identified as a potential predictor for adverse cardiovascular events in patients with coronary heart disease. The study by Mone et al*.* [24] showed that the risk of re-hospitalization with chest pain is significantly increased in patients with ischemia and nonobstructive coronary arteries who have a SHR > 1 for 1 year, suggesting that the SHR may be used as an independent risk factor for these patients. Additionally, the SHR has been identified as a potential predictor for death in patients with MI, especially in those without DM [7]. The possible reasons are as follows: (1) chronic hyperglycemia may protect the myocardium via hypoxia-induced apoptosis and necrosis [25]; (2) the prognosis of DM patients with MI is poor. In addition to hyperglycemia, the influence of diabetic cardiomyopathy should also be excluded [26]; and (3) diabetic patients are more often treated with insulin and other hypoglycemic drugs. Previous studies have confirmed that insulin can reduce the mortality of patients with AMI [27]. Nondiabetic patients with STEMI tend to have a worse prognosis than those with DM. The HORIZONS-AMI trial, a prospective study of 3,405 STEMI patients undergoing direct PCI, reported a positive correlation between acute hyperglycemia and mortality in the nondiabetic subgroup, which was stronger than that in the DM subgroup [28]. These data imply that the effect of acute hyperglycemia might be masked in diabetic patients because DM itself may lead to poor long-term prognosis. It may also be explained by more severe inflammation in patients with newly diagnosed hyperglycemia than those with known DM [29]. In our subgroup analysis, patients with previously diagnosed DM or significantly elevated HbA1c were excluded, and the effects of stress hyperglycemia on in-hospital outcomes in nondiabetic patients were investigated. The results showed that SHR was also a predictor of MACCE in the nondiabetic subgroup.

Currently, there is no consensus on the definition and reference interval for stress hyperglycemia. In 2015, Roberts et al*.* [5] enrolled 2, 290 patients admitted to the emergency department and investigated whether critical illness was correlated with relative or absolute hyperglycemia. In their univariate analysis, both the SHR (OR = 1.23 mmol/L; 95% CI: 1.18–1.28; *P* < 0.001) and Glu (OR = 1.18 mol/L; 95% CI: 1.13–1.23; *P* < 0.001) were independent risk factors for critical illness. In their multivariate analysis, however, only SHR [OR = 1.20 95% CI: 1.13–1.28; *P* < 0.001] remained significantly correlated, while Glu [OR = 1.03 mmol/L (0.97, 1.11); *P* = 0.31] did not.

The relationship between the SHR and the prognosis of STEMI patients has been previously investigated. In a retrospective study of 4,362 patients with coronary heart disease who underwent PCI in the COACT Registry, the SHR was identified as a predictor for MACCE within 30 days after PCI, especially in nondiabetic patients [6]. A recent prospective, multicenter observational study of 6,287 STEMI patients who were discharged alive showed that a high SHR was significantly correlated with poor long-term prognosis in the nondiabetic population, but not in the diabetic population [29]. Additionally, Chen et al*.* reported the potential of the SHR as an independent and simple indicator of poor in-hospital prognosis in elderly patients with AMI, especially in the nondiabetic population [30]. The above data are consistent with the results of the present study.

The limitations of this work should also be acknowledged. Firstly, this was a single-center study with a limited sample size. Secondly, due to the retrospective nature of the study, long-term observation and follow-up after discharge were not performed. Further studies with a prospective design and long-term follow-up are needed. Thirdly, stress may impair the homeostasis of many systems, including the renin–angiotensin–aldosterone system, the sympathetic system, and the inflammatory system, which may ultimately affect in-hospital outcomes.

## Conclusion

In conclusion, the SHR, as a new parameter of stress-induced hyperglycemia, is an independent predictor of MACCE in patients with STEMI, especially in the nondiabetic population. The SHR can be easily calculated from HbA1c and Glu, and therefore, can be widely applied in clinical practice for early risk stratification of newly diagnosed STEMI patients.

## Data Availability

The datasets generated and analyzed during the current study are not publicly available due to limitations of ethical approval involving the patient data and anonymity but are available from the corresponding author on reasonable request.

## Abbreviations

SHR: Stress hyperglycemia ratio
MACCE: Major adverse cardiovascular and cerebrovascular events
STEMI: ST-segment elevation myocardial infarction
OR: Odds ratio
DM: Diabetes mellitus
AMI: Acute myocardial infarction
HbA1c: Glycated hemoglobin
PSM: Propensity score matching
cTn: Cardiac troponin
MI: Myocardial infarction
DBP: Diastolic blood pressure
SBP: Systolic blood pressure
HR: Heart rate
IABP: Intra-aortic balloon pump
WBC: White blood cell
CRP: C-reactive protein
Cr: Serum creatinine
BNP: Brain natriuretic peptide
LVEF: Left ventricular ejection fraction
PCI: Percutaneous coronary intervention
CI: Confidence interval
CKD: Chronic kidney disease
PAD: Peripheral arterial disease
CABG: Coronary artery bypass grafting
LM: Left main coronary artery
LAD: Left anterior descending coronary artery
LCX: Left circumflex coronary artery
RCA: Right coronary artery
RR: Respiratory rate
HGB: Hemoglobin
PLT: Blood platelet count
ALT: Alanine transaminase
ALB: Albumin
AST: Aspartate aminotransferase
Glu: Blood glucose
TG: Total triglycerides
TC: Total cholesterol
HDL-C: High-density lipoprotein cholesterol
LDL-C: Low-density lipoprotein cholesterol
CK: Creatine kinase
LDH: Lactate dehydrogenase
K: Serum potassium
LVEDD: Left ventricular end-diastolic dimension
S.E: Standard error

## References

[1] Reed GW, Rossi JE, Cannon CP (2017). Acute myocardial infarction. Lancet.

[2] Lundberg V, Stegmayr B, Asplund K, Eliasson M, Huhtasaari F (1997). Diabetes as a risk factor for myocardial infarction: population and gender perspectives. J Intern Med.

[3] Monteiro S, António N, Gonçalves F, Monteiro P, Freitas M, Providência LA (2009). Glycemia at admission: the metabolic echocardiography in acute coronary syndrome patients. Eur J Cardiovasc Prev Rehabil.

[4] Chen PC, Chua SK, Hung HF, Huang CY, Lin CM, Lai SM (2014). Admission hyperglycemia predicts poorer short- and long-term outcomes after primary percutaneous coronary intervention for ST-elevation myocardial infarction. J Diabetes Investig.

[5] Roberts GW, Quinn SJ, Valentine N, Alhawassi T, O’Dea H, Stranks SN, et al. Relative hyperglycemia, a marker of critical illness: introducing the stress hyperglycemia ratio. J Clin Endocrinol Metab. 2015;100:4490–7. 10.1210/jc.2015-2660 26485219

[6] Khalfallah M, Abdelmageed R, Elgendy E, Hafez YM (2020). Incidence, predictors and outcomes of stress hyperglycemia in patients with ST elevation myocardial infarction undergoing primary percutaneous coronary intervention. Diab Vasc Dis Res.

[7] Capes SE, Hunt D, Malmberg K, Gerstein HC (2000). Stress hyperglycaemia and increased risk of death after myocardial infarction in patients with and without diabetes: a systematic overview. Lancet.

[8] Egi M, Bellomo R, Stachowski E, French CJ, Hart GK, Taori G (2011). The interaction of chronic and acute glycemia with mortality in critically ill patients with diabetes. Crit Care Med.

[9] Thygesen K, Alpert JS, Jaffe AS, Chaitman BR, Bax JJ, Morrow DA (2018). Fourth universal definition of myocardial infarction (2018). J Am Coll Cardiol.

[10] American Diabetes Association Professional Practice Committee (2022). 2 classification and diagnosis of diabetes: standards of medical care in diabetes-2022. Diabetes Care.

[11] Horne BD, May HT, Muhlestein JB, Ronnow BS, Lappé DL, Renlund DG (2009). Exceptional mortality prediction by risk scores from common laboratory tests. Am J Med.

[12] Mertİlker H, Faysal S, Ahmet Çağdaş Y, Murat S, Tufan Ç (2023). Prognostic value of Intermountain Risk Score for short- and long-term mortality in patients with cardiogenic shock. Coron Artery Dis.

[13] Çınar T, Şaylık F, Akbulut T, Korkmaz Y, Çiçek V, Asal S (2023). Evaluation of Intermountain risk score for short- and long-term mortality in ST elevation myocardial infarction patients. Angiology.

[14] Paolisso P, Foà A, Bergamaschi L, Angeli F, Fabrizio M, Donati F (2021). Impact of admission hyperglycemia on short and long-term prognosis in acute myocardial infarction: MINOCA versus MIOCA. Cardiovasc Diabetol.

[15] Hayıroğlu M, Keskin M, Uzun AO, Yıldırım D, Kaya A, Çinier G (2019). Predictors of in-hospital mortality in patients with ST-segment elevation myocardial infarction complicated with cardiogenic shock. Heart Lung Circ.

[16] Hayıroğlu M, Çanga Y, Yıldırımtürk Ö, Bozbeyoğlu E, Gümüşdağ A, Uzun AO (2018). Clinical characteristics and outcomes of acute coronary syndrome patients with intra-aortic balloon pump inserted in intensive cardiac care unit of a tertiary clinic. Turk Kardiyol Dern Ars.

[17] Chu J, Tang J, Lai Y, Gao Y, Ye Z, Guan C (2020). Association of stress hyperglycemia ratio with intracoronary thrombus burden in diabetic patients with ST-segment elevation myocardial infarction. J Thorac Dis.

[18] Ujueta F, Weiss EN, Sedlis SP, Shah B (2016). Glycemic control in coronary revascularization. Curr Treat Options Cardiovasc Med.

[19] Timmer JR, Hoekstra M, Nijsten MW, van der Horst IC, Ottervanger JP, Slingerland RJ (2011). Prognostic value of admission glycosylated hemoglobin and glucose in nondiabetic patients with ST-segment-elevation myocardial infarction treated with percutaneous coronary intervention. Circulation.

[20] Eitel I, Hintze S, de Waha S, Fuernau G, Lurz P, Desch S (2012). Prognostic impact of hyperglycemia in nondiabetic and diabetic patients with ST-elevation myocardial infarction: insights from contrast-enhanced magnetic resonance imaging. Circ Cardiovasc Imaging.

[21] Esposito K, Nappo F, Marfella R, Giugliano G, Giugliano F, Ciotola M (2002). Inflammatory cytokine concentrations are acutely increased by hyperglycemia in humans: role of oxidative stress. Circulation.

[22] Mifsud S, Schembri EL, Gruppetta M (2018). Stress-induced hyperglycaemia. Br J Hosp Med (Lond).

[23] Ceriello A. Acute hyperglycaemia: a “new” risk factor during myocardial infarction. Eur Heart J. 2005;26:328–31. 10.1093/eurheartj/ehi049 15618047

[24] Mone P, Lombardi A, Salemme L, Cioppa A, Popusoi G, Varzideh F (2023). Stress hyperglycemia drives the risk of hospitalization for chest pain in patients with Ischemia and Nonobstructive Coronary Arteries (INOCA). Diabetes Care.

[25] Schaffer SW, Croft CB, Solodushko V (2000). Cardioprotective effect of chronic hyperglycemia: effect on hypoxia-induced apoptosis and necrosis. Am J Physiol Heart Circ Physiol.

[26] Jankauskas SS, Kansakar U, Varzideh F, Wilson S, Mone P, Lombardi A (2021). Heart failure in diabetes. Metabolism.

[27] Malmberg K, Norhammar A, Wedel H, Rydén L (1999). Glycometabolic state at admission: important risk marker of mortality in conventionally treated patients with diabetes mellitus and acute myocardial infarction: long-term results from the Diabetes and Insulin-Glucose Infusion in Acute Myocardial Infarction (DIGAMI) study. Circulation.

[28] Planer D, Witzenbichler B, Guagliumi G, Peruga JZ, Brodie BR, Xu K (2013). Impact of hyperglycemia in patients with ST-segment elevation myocardial infarction undergoing percutaneous coronary intervention: the HORIZONS-AMI trial. Int J Cardiol.

[29] Kojima T, Hikoso S, Nakatani D, Suna S, Dohi T, Mizuno H (2020). Impact of hyperglycemia on long-term outcome in patients with ST-segment elevation myocardial infarction. Am J Cardiol.

[30] Chen G, Li M, Wen X, Wang R, Zhou Y, Xue L (2021). Association between stress hyperglycemia ratio and in-hospital outcomes in elderly patients with acute myocardial infarction. Front Cardiovasc Med.

